# Correction: Protein Phosphatase 2A as a Therapeutic Target in Small Cell Lung Cancer

**DOI:** 10.1158/1535-7163.MCT-22-0104

**Published:** 2022-04-04

**Authors:** Tamara Mirzapoiazova, Gang Xiao, Bolot Mambetsariev, Mohd W. Nasser, Emily Miaou, Sharad S. Singhal, Saumya Srivastava, Isa Mambetsariev, Michael S. Nelson, Arin Nam, Amita Behal, Leonidas D. Arvanitis, Pranita Atri, Markus Muschen, François L.H. Tissot, James Miser, John S. Kovach, Martin Sattler, Surinder K. Batra, Prakash Kulkarni, Ravi Salgia

In the original version of this article (1), author, Leonidas D. Arvanitis, is mistakenly omitted from the author list. This error has been corrected in the latest online HTML and PDF versions of the article and the author contributions and disclosures have been updated as necessary. The authors regret this error.
